# Integrative analysis of the nuclear proteome in *Pinus radiata* reveals thermopriming coupled to epigenetic regulation

**DOI:** 10.1093/jxb/erz524

**Published:** 2019-11-29

**Authors:** Laura Lamelas, Luis Valledor, Mónica Escandón, Gloria Pinto, María Jesús Cañal, Mónica Meijón

**Affiliations:** 1 Plant Physiology, Department of Organisms and Systems Biology, Faculty of Biology and Biotechnology Institute of Asturias, University of Oviedo, Oviedo, Asturias, Spain; 2 Department of Biology and CESAM, University of Aveiro, Aveiro, Portugal; 3 RWTH Aachen University, Germany

**Keywords:** Adaptation, epigenetic memory, high-temperature stress, omics approach, *Pinus*, priming

## Abstract

Despite it being an important issue in the context of climate change, for most plant species it is not currently known how abiotic stresses affect nuclear proteomes and mediate memory effects. This study examines how *Pinus radiata* nuclei respond, adapt, ‘remember’, and ‘learn’ from heat stress. Seedlings were heat-stressed at 45 °C for 10 d and then allowed to recover. Nuclear proteins were isolated and quantified by nLC-MS/MS, the dynamics of tissue DNA methylation were examined, and the potential acquired memory was analysed in recovered plants. In an additional experiment, the expression of key gene genes was also quantified. Specific nuclear heat-responsive proteins were identified, and their biological roles were evaluated using a systems biology approach. In addition to heat-shock proteins, several clusters involved in regulation processes were discovered, such as epigenomic-driven gene regulation, some transcription factors, and a variety of RNA-associated functions. Nuclei exhibited differential proteome profiles across the phases of the experiment, with histone H2A and methyl cycle enzymes in particular being accumulated in the recovery step. A thermopriming effect was possibly linked to H2A abundance and over-accumulation of spliceosome elements in recovered *P. radiata* plants. The results suggest that epigenetic mechanisms play a key role in heat-stress tolerance and priming mechanisms.

## Teaser

Heat stress followed by a recovery period leads to changes in the nuclear proteome of *Pinus radiata* that suggest that epigenetic mechanisms play a key role in tolerance and priming mechanisms.

## Introduction

Forests provide the world’s population with many far-reaching benefits. Among many other things, these include wood supplies and socio-economic goods, the promise of mid-term mitigation of increasing atmospheric CO_2_ concentrations, and the delivery of vital long-term environmental benefits such as clean air, water, and biodiversity (FAO, 2015).

*Pinus radiata* is currently the most widely planted pine species for forestry due to its fast growth, acceptable wood quality, and economically profitable production (Mead, 2013). According to numerous climate models and, increasingly, observations, temperatures are rising, and heatwave events are becoming more frequent. Within this context of climate change and coupled with an increasing demand for wood (FAO, 2009), there is a clear need to study the adaptation mechanisms of species such as *P. radiata* to heat stress, especially as high temperature is one of the most detrimental stresses that limits the growth of temperate forest trees (Wahid *et al.*, 2007).

Heat-stress responses involve changes in multiple mechanisms and metabolic pathways. Although a number of the more important players have been described in *P. radiata*, such as heat shock proteins, flavonoids, and fatty acids (Escandón *et al.*, 2016, 2017, 2018), it is still largely unknown how gene regulation is changed in order to bring about these biochemical and physiological responses and adaptations.

Knowledge of nuclear proteome dynamics is crucial for increasing our understanding of how both environmental and cytoplasmic signals are sensed and translated into molecular responses, which is mainly through the proteins that guide and control gene expression. Nuclear proteomics provides a useful approach not just for investigating the mechanisms underlying plant responses to abiotic stresses, such as protein–protein interactions, enzyme activities, and post-translational modifications (Yin and Komatsu, 2016), but also for potentially creating solutions to improve forest management and breeding programmes.

In *P. radiata*, metabolic rearrangements as a consequence of exposure to UV radiation occur through specific regulation of chromatin dynamics and gene expression by histones and nuclear factor-Y transcription factors (NF-Ys) (Pascual *et al.*, 2016). Alternative splicing and other RNA translation and protein synthesis proteins also play an important role; however, there is no indication of how pines remember this stress.

Nuclear post-transcriptional regulatory mechanisms play an important role in relation to heat-stress memory in Arabidopsis and involve the processing of precursor mRNA (alternative splicing) (Ling *et al.*, 2018) as well as structural variations in the histone H2A and H2B dimers of the nucleosome (Talbert and Henikoff, 2014). Nevertheless, epigenetic factors are thought to play the main role in establishing this heat-stress memory (Ling *et al.*, 2018).

In this respect, and in relation to the relevance of nuclear proteins in driving heat-stress adaptation, the key roles of epigenetic regulation and histone modifications in retaining memory of the stress (Bäurle, 2016; Lämke *et al.*, 2016) that leads to the priming mechanism (Martinez-Medina *et al.*, 2016) has been described in Arabidopsis. Priming involves a first ‘training’ stress, a latent phase, and then a second stress event, in which the plant will be able to react in a more efficient way than previously, due to the information stored as chromatin structural changes and histone modifications (Gutzat and Mittelsten Scheid, 2012; Pastor *et al.*, 2013; Asensi-Fabado *et al.*, 2017). The epigenetic mechanisms, particularly DNA methylation and nucleosome occupancy, seem to be the main players in establishing priming. The former involve covalent modifications of DNA and histones, which affect the transcriptional activity of chromatin (Valledor *et al.*, 2007). Since chromatin states can be propagated through cell division, epigenetic mechanisms are thought to provide heritable ‘cellular memory’ (Iwasaki and Paszkowski, 2014).

This study aimed to provide comprehensive data for the heat-stress response and adaptation at nuclear level that will allow determination of the nuclear events involved in heat-stress memory. The main goals were the characterization, quantification, and biological interpretation of the nuclear proteome of *P. radiata* needles in response to high-temperature stress and an assessment of the recovery stage (Phase I) using an experimental design that simulated a realistic temperature increase scenario. The dynamics of DNA methylation at the tissue level were also analysed. In addition, a second round of stress was imposed (Phase II), and key elements and pathways related to thermopriming processes were identified and validated by qPCR, and the responses to the first and second stress exposures were compared. The identification of proteins related to chromatin reorganization provides a major advance in the field of heat-stress biology, and our data also provide a set of key nuclear elements in cytoplasmic proteome reorganization associated with the heat-stress response and thermopriming process.

## Materials and methods

### Plant material

Six-month-old seedlings of *Pinus radiata* D. Don (height 22±4 cm) in 1-dm^3^ pots were kept in a climate chamber (Fitoclima 1200, Aralab) under a photoperiod of 16 h (400 μmol m^−2^ s^−1^) at 25 °C and 50% relative humidity (RH), and 8 h at 15 °C and 60% RH during the night period. The plants had been previously acclimated over a 1-month period inside the chamber, and were watered with nutritive solution (N : P : K 5 : 8 : 10).

### Experimental design

In order to establish the optimal temperature for the heat-stress assay, mature *P. radiata* needles were exposed *ex vivo* to different temperatures (25, 40, 42, 45, 48, and 50 °C) as described by Ferreira *et al.* (2006) with the modifications of Escandón *et al.* (2016). Electrolyte leakage (EL) was used to determine cell membrane damage (see below) as a marker of plant performance under stress. Based on the preliminary results of plant tolerance, a maximum temperature of 45 °C for a period of 10 d was selected for the treatment (Supplementary Fig. S1 at *JXB* online).

Before starting the experiment, plants were divided in two sets. Set I was stressed twice, in experimental Phases I and II, while Set II was stressed only in Phase II to test whether there was any long-lasting memory acquisition related to a priming process.

**Fig. 1.**
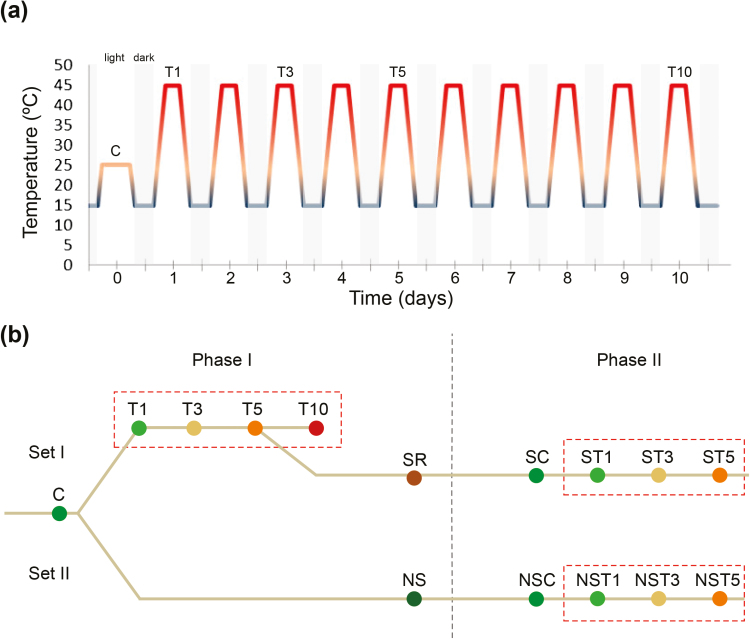
Experimental design. (a) Detailed temperature profile of control and heat-stress conditions to which seedlings of *Pinus radiata* were subjected. The control condition temperatures were set to 25 ºC during the day and to 15 ºC at night. Heat exposure began each day of the stress treatment with an increasing temperature gradient from 15 °C to 45 °C over a period of 5 h, and this was then maintained for 6 h. Over the following 5 h the temperature was returned to 15 ºC and maintained for 8 h, thus mimicking a day–night scenario. (b) Outline of the experimental set-up. Control plants were sampled and then divided in two sets. In Phase I, Set I plants were stressed and sampled at intervals up to 10 d to characterize the responses: T1, T3, T5, and T10, are as shown in (a). Then, stressed plants from the T5 treatment were kept in control conditions and carried forward to Phase II. Meanwhile, Set II plants were allowed to grow in control conditions. Both sets were sampled 1 month after the first round of stress in order to evaluate whether previously stressed plants developed mid-term memory acquisition: SR, stress-recovered, belonging to Set I; and NS, not stressed, belonging to Set II. At 6 months after the end of Phase I, the plants were subjected to another round of treatment (Phase II). Both sets were subjected to heat stress for up to 5 d. SC: previously stressed plants, control; ST1, ST3, ST5: previously stressed plants sampled after 1, 3, or 5 d of stress, as shown in (a). NS indicates plants that were not previously stressed (i.e. Set II). (This figure is available in colour at *JXB* online.)

In Phase I, the Set I seedlings were sampled under control and then heat-stressed conditions according to the procedure detailed in Fig. 1a. Plant material was sampled at the end of the 6-h heat exposure on day 1 (T1), day 3 (T3), day 5 (T5), and day 10 (T10). Since T10 plants were clearly too damaged, T5 plants were selected to continue to Phase II. The T5 and Set II seedlings were maintained under control conditions and sampled after 1 month, when the plants showed an apparent physiological recovery and possible intermediate memory (Fig. 1b; SR, stress-recovered and NS, not stressed). Needles were frozen in liquid nitrogen immediately after sampling and stored at −80 °C until isolation of the nucleus and proteins was performed. Sections of needles were also fixed in 4% paraformaldehyde for further 5-methyldeoxycytidine (5-mdC) immunolocalization analysis. Gas-exchange parameters were quantified just prior to sampling, and cell membrane damage was measured in fresh needles immediately after sampling by quantifying relative EL (see below).

To test for possible long-term stress memory, 6 months later in Phase II a second round of heat stress was performed for 5 d in both sets of plants according to the same scheme (Fig. 1b). For each set, sampling was performed for controls and for plants heat-treated for 1, 3, or 5 d. Chlorophyll fluorescence measurements were taken just prior to sampling (see below), as photosynthesis activity was found to be a clear marker of plant health status upon heat stress.

In both sets, plants were divided into four pools, constituted of needles of three plants each. These pools were kept across the experiment and formed the four independent biological replicates that were analysed.

### Gas-exchange measurements

Net CO_2_ assimilation rate (*A*), stomatal conductance (*g*_s_), transpiration rate (*E*), and intercellular CO_2_ concentration (*C*_i_) were measured in all plants and averaged for each biological replicate per treatment in Phase I. A portable infrared gas analyser (LCpro-SD, ADC BioScientific Ltd, UK) equipped with a specialized needle chamber was used. Light-response curves of CO_2_ assimilation were obtained with the following values of photosynthetic photon flux densities (PPFDs): 2000, 1500, 1000, 750, 500, 250, 100, 50, and 0 µmol m^−2^ s^−1^. After analysis of the *A*/PPFD data, measurements at saturation light intensity were performed at 1000 µmol m^−2^ s^−1^. The following conditions were maintained inside the chamber during all measurements: air flux, 200 mol s^−1^; block temperature, 25 °C; and atmospheric CO_2_ and H_2_O concentrations. Data were recorded when the measured parameters were stable. Instantaneous carboxylation efficiency (CE) was calculated as the coefficient of *A* and *C*_i_.

### Cell membrane damage

The percentage of EL was used to determine cell membrane damage in samples from Phase I during the heat-stress treatments (C, T1, T3, T5, and T10; Fig. 1a) and in the preliminary experiment to establish the optimal treatment temperature. Needles (100 mg) of each biological replicate were collected at each sampling point, cut into 1 cm-long pieces, immediately immersed in sterile de-ionized water, and then incubated for 24 h at room temperature under agitation at 30 rpm on a shaker (Ch-4103; Infors HT). The conductivity of the water was then measured (*Con*_sample_) using a conductivity meter (sensION +MM150 portable meter, Hach). The conductivity of the de-ionized water without any sample present was also measured (*Con*_i_). After autoclaving for 20 min at 1100 KPa and 121 °C, followed by cooling at room temperature overnight under agitation, the maximum conductivity (*Con*_max_) was measured. Again, the conductivity of the de-ionized water without any sample present was also measured (*Con*_ii_). The percentage of EL was then calculated as EL(%) = [(*Con*_sample_ − *Con*_i_)/(*Con*_max_ − *Con*_ii_)] × 100.

### Nucleus isolation and protein extraction

The nuclear proteome was analysed for the four biological replicates of Phase I at all sampling times (C, T1, T3, T5, T10, NS, and SR; Fig. 1b). Nuclei were isolated following the protocol described by Alegre *et al.* (2016). Isolation and enrichment efficiency were assessed by confocal microscopy (Leica TCS-SP2-AOBS) using propidium iodide dye (Supplementary Fig. S2). The dye was excited by a diode laser at 488 nm and the fluorescence emission was recorded at 636 nm. Images were processed and analysed using the Fiji software (Schindelin *et al.*, 2012).

Once the nuclei were purified, samples were sonicated in 300 μl of 1% SDS for 15 s at 60% amplitude (Hielscher UP200S) and then incubated in a vortex at maximum speed (2500 rpm) for 15 min at room temperature. Then 300 μl of extraction buffer (1.5 M sucrose, 10 mM DTT, and 300 μl of phenol) was added to begin the protein extraction. After mixing vigorously, tubes were centrifuged for 5 min at 17 000 *g* at room temperature. After centrifugation, the phenolic (upper) phase was saved and the lower phase was re-extracted by adding 300 μl of phenol. The two phenolic phases were combined, 300 μl of extraction buffer was added, the solution was mixed and centrifuged again as described above, and the upper phase was saved. Proteins were precipitated by adding 0.1 M ammonium acetate in methanol and incubating overnight at −20 °C. The tubes were centrifuged, and the protein pellets were washed twice with acetone. The dry pellets were dissolved in 1.5% SDS and 8 M urea. The protein content was quantified using a BCA assay (Smith *et al.*, 1985). Enrichment in the nuclear proteins was assessed by comparing the running nuclear protein fraction and the total protein in one-dimensional (1D) SDS-PAGE.

### Protein identification and quantitation by GeLC-Orbitrap/MS analysis

Proteins were gel-digested with trypsin (Roche, cat. no. 03 708 969 001) and the peptides obtained were extracted and desalted as described by Valledor and Weckwerth (2014).

The peptides were analysed by the University of Cordoba Central Support Service for Research (SCAI) using a 1D nano-flow LC coupled to an MS/MS Orbitrap Fusion spectrometer (ThermoFisher Scientific), using a 60-min gradient starting with 0.1% formic acid and with 80% acetonitrile as the mobile phase.

Three protein databases were used for protein identification, namely *Pinus taeda* genome v.1.01 (https://bioinformatics.psb.ugent.be/plaza/versions/gymno-plaza/), UniProt/SwissProt Viridiplantae, and an in-house *P. radiata* transcriptome (unpublished), following the recommendations described by Romero-Rodríguez *et al.* (2014).

Proteome Discoverer 2.2 was used for the identification and quantification of proteins, employing a 2% false discovery rate (FDR), XCorr of 1.6, one unique or razor peptide for identification, and one peptide (unique/razor) per protein for label-free quantification. Lysine ubiquitination, methionine oxidation, acetylation of the protein N-terminus, and phosphorylation of serine, threonine, and tyrosine were taken into account as dynamic modifications.

Identified protein sequences were analysed with the PlantTFcat tool (Dai *et al.*, 2013) to identify transcription factors and nuclear regulator domains, together with two independent and plant-specific subcellular localization tools, Localizer v1.0.4. (Sperschneider *et al.*, 2017) and YLoc (Briesemeister *et al.*, 2010*a*, 2010*b*) using the YLoc+ prediction model and plant version with a fixed probability greater than 0.75 and medium confidence score (at least 0.4). Proteins were also annotated using the Mercator sequence annotation tool against the TAIR, SwissProt/UniProt Plant Proteins, and Clusters of orthologous eukaryotic genes (KOG) databases (Lohse *et al.*, 2014) and UniProt KB/SwissProt using sma3s v2 (Casimiro-Soriguer, 2017). Restrictive conditions were used to catalogue nuclear proteins. Proteins not predicted to belong to the nucleus or endoplasmic reticulum by at least of two of the five selected data sources were dropped from the analysis.

### Immunolocalization of 5-mdC

Methylated DNA was monitored in samples C, T1, T3, T5, and SR (Fig. 1b) by 5-mdC immunolocalization according to the procedure described by Meijón *et al.* (2010). Briefly, fixed needles were sectioned at 50 µm thickness using a CH 1510-1 cryomicrotome (Leica Microsystems). The samples were permeabilized, blocked with bovine serum albumin and incubated with anti-5-mdC mouse antibody (Eurogentec, Belgium) diluted 1 : 50 in 1% blocking solution. Alexa Fluor 488-labelled anti-mouse polyclonal antibody (Invitrogen) diluted 1 : 25 was used as secondary antibody for detection of 5-mdC. The slides were counterstained with DAPI. Fluorescence was visualized using a TCS-SP2-AOBS confocal microscope (Leica). Maximal projection from a stack of six slides per sample was acquired using the Fiji software (Schindelin *et al.*, 2012).

### Chlorophyll fluorescence, and total contents of soluble sugars and phenolic compounds

Chlorophyll fluorescence kinetics were measured on needles of all the plants in Phase II of the experiment using a pulse-amplitude modulation fluorimeter (Mini-PAM, Walz) according to Jesus *et al.* (2015). For the same plants, total contents of soluble sugars (TSS) and phenolic compounds were determined using 10 mg of lyophilized samples following the anthrone and Folin–Ciocalteu methods, respectively, as described by Jesus *et al.* (2015).

### Transcriptomic study for candidate genes using qPCR

RNA was extracted from four biological replicates of each treatment of Phase II according to Valledor *et al.* (2014) and then quantified in a spectrophotometer. RNA integrity was checked by agarose gel electrophoresis, and for potential DNA contamination by PCR employing the ubiquitin (UBI) primer pair.

Then 500 ng of RNA was reversed-transcribed using a RevertAid kit (ThermoFisher Scientific) and random hexamers as primers following the manufacturer’s instructions.

qPCR reactions were performed in a CFX Connect Real Time PCR machine (Bio-Rad) with SsoAdvanced Universal SYBR Green Supermix (Bio-Rad); three biological and three analytical replicates were used.

Normalized relative quantities (NRQ) and standard errors of RQ were determined according to Hellemans (2007). Expression levels of actin (ACT) and GAPDH (GAPDH) were used as endogenous controls. Details of the primers used for qPCR are available in Supplementary Table S1.

### Statistical and bioinformatics analyses

All statistical procedures were conducted using R running under the open-source computer software R v3.4.0 (www.r-project.org) and RStudio v1.1.456 (http://www.rstudio.org/).

Four biological replicates were used for all statistical procedures. The agricolae package (http://CRAN.R-project.org/package=agricolae) was used for univariate ANOVA followed by *post hoc* multiple comparisons using Tukey’s test (function HSD.test) to estimate the significance of the leaf gas-exchange and membrane damage data.

The nuclear proteome was analysed using the pRocessomics R package (https://github.com/Valledor/pRocessomics) developed in our lab, which was used to pre-filter and impute missing values according to the Random Forest algorithm in the missForest package (Stekhoven and Bühlmann, 2012) and consistency-based criteria with a threshold of 0.25. The three most stable protein abundances were then identified with the sLqPCR package according to Vandesompele *et al.* (2002), and their mean values were used for normalizing each sample to equalize inter-sample variations in total intensity.

The most significant nuclear proteins were selected by ANOVA with a cut-off *q*-value of 0.05 calculated using the Benjamini–Hochberg model. Then, the mixOmics R package (Rohart *et al.*, 2017) was used to perform statistical analyses, including principal component analysis (PCA) and sparse partial least-square (sPLS) analysis. This provided two networks, the first of which integrated physiological and proteomics data, and the second identified transcription factors with significantly different nuclear proteins. Both networks were filtered by applying a net cut-off of 0.75.

## Results

### High temperature affects gas-exchange parameters and membrane integrity

**Fig. 2.**
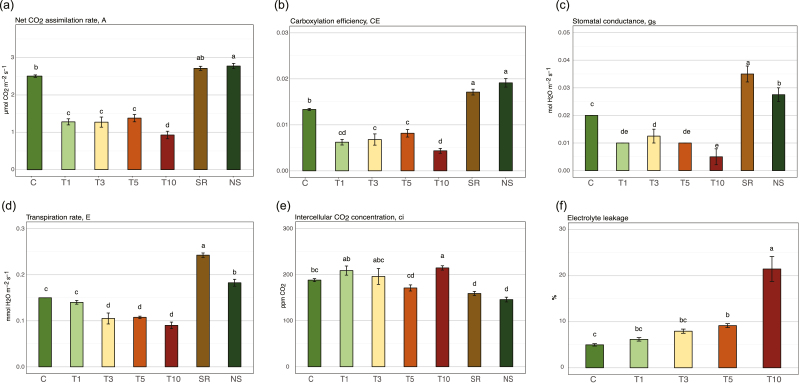
Gas-exchange parameters and electrolyte leakage in needles of *Pinus radiata* seedlings subjected to heat stress. (a) Net CO_2_ assimilation rate, *A*; (b) carboxylation efficiency, CE; (c) stomatal conductance, *g*_s_; (d) transpiration rate, *E*; (e) intercellular CO_2_ concentration, *C*_i_; and (f) electrolyte leakage, EL. The measurements were taken during Phase I of the experiment, as shown in Fig. 1. Data are means (±SE), *n*=4. Different letters indicate significate differences as determined using ANOVA followed by Tukey’s HSD test (*P*<0.05). (This figure is available in colour at *JXB* online.)

Analysis of leaf gas-exchange parameters during experimental Phase I (Fig. 1b) identified significant physiological changes in response to heat stress. Stressed plants showed significant decreases in net CO_2_ assimilation rate (*A*), carboxylation efficiency (CE), stomatal conductance (*g*_s_), and transpiration rate (*E*) (Fig. 2a–d). The effects were more severe when the stress exposure time was increased, and showed a similar pattern in the four parameters. This, coupled with maintenance of the intercellular CO_2_ content (Fig. 2e), suggested a decrease of carbon fixation in the stressed plants in favour of photorespiration at sampling time T10, when the intercellular CO_2_ concentration (*C*_i_) was increased. In addition, the electrolyte leakage (EL) showed significant differences at T5 and it increased dramatically at T10, indicating severe disruption of cell membrane integrity (Fig. 2f).

After a 1-month period of recovery in control conditions, stressed-recovered (SR) and non-stressed (NS) plants presented similar CO_2_ values of *A* and CE (Fig. 2a, b), indicating that photosynthetic activity returned to normal. On the other hand, higher values of *g*_s_ and *E* (Fig. 2c, d) were observed in recovered stressed plants, which were probably related to some adaptation process or memory effect.

An age effect was also detected when comparing the initial control and NS groups, with increases in *E*, *g*_s_, and CE and a decrease in *C*_i_ suggesting different physiological states that were possibly related to plant growth during the recovery period.

### Identification and characterization of nuclear proteins across periods of exposure to heat stress

GeLC-Orbitrap/MS analysis of the nuclei-enriched fraction resulted in the identification of 3571 protein groups, of which 3328 could be reliably quantified. Given the limitations of non-model databases, very restrictive conditions were used for classifying nuclear proteins. Thus, after removal of the non-nuclear proteins based on their *in silico* annotations and YLoc and Localizer subcellular localization tools, 862 protein groups were identified as certainly being nuclear, of which 309 (*q*-value ≤0.05) were considered as differentially accumulated (Supplementary Table S2; ANOVA, 5% FDR).

**Fig. 3.**
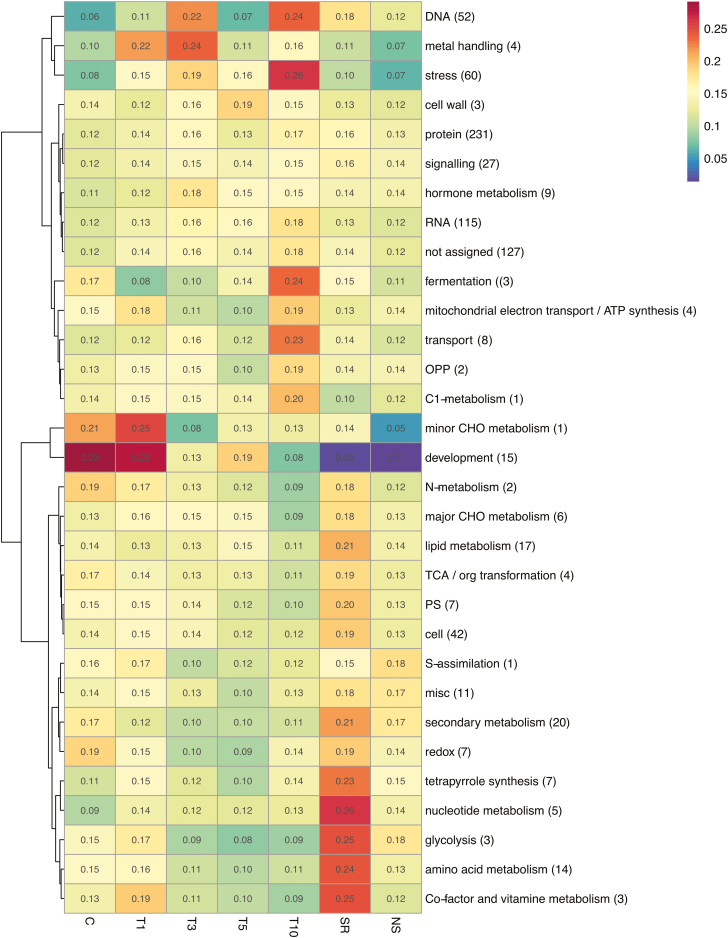
Heatmap-clustering analysis using MapMan categorization pathways in the nuclear proteome of needles of *Pinus radiata* seedlings subjected to heat stress. The numbers indicate the scaled abundance according to the MapMan functional bin. Manhattan distance and Ward’s aggregation method were used for hieratical clustering. The numbers of proteins included in each category are indicated. The sampling times correspond to the experimental set-up shown in Fig. 1.

MapMan functional classification of the identified nuclear proteins showed that the differentially accumulated pathways covered both primary and secondary metabolism (Fig. 3). The abundance of stress-related clusters (stress and metal handling) increased under heat-stress conditions. In addition, T5 seedlings showed signs of recovery as their values for development and cell wall clusters showed increases. However, in T10 samples the development, cell wall, and protein metabolism clusters were found to be down-regulated; moreover, these samples showed considerable increases in fermentation and stress-related clusters, which highlighted cell impairment consistent with the physiological results (Fig. 2a–f).

After the recovery period, SR plants exhibited differences compared to unstressed plants. Nucleotide, amino acid, cofactor and vitamin, and secondary metabolism were clearly up-regulated, as were the tetrapyrrole synthesis and glycolysis pathways.

There were considerable changes in the development category between the C–T1 and the SR–NS groups; however, these seemed to be linked to the age of the plants rather than being relevant for analysing the heat-stress response. This highlighted the importance of maintaining control plants throughout the experiment.

In summary, the functional classification and heatmap clustering analysis of the nuclear proteome distinguished the different periods of heat exposure and allowed the determination of specific pathway clusters related to each exposure period.

### A multivariate approach reveals the nuclear mechanisms involved in the heat-stress and memory responses

**Fig. 4.**
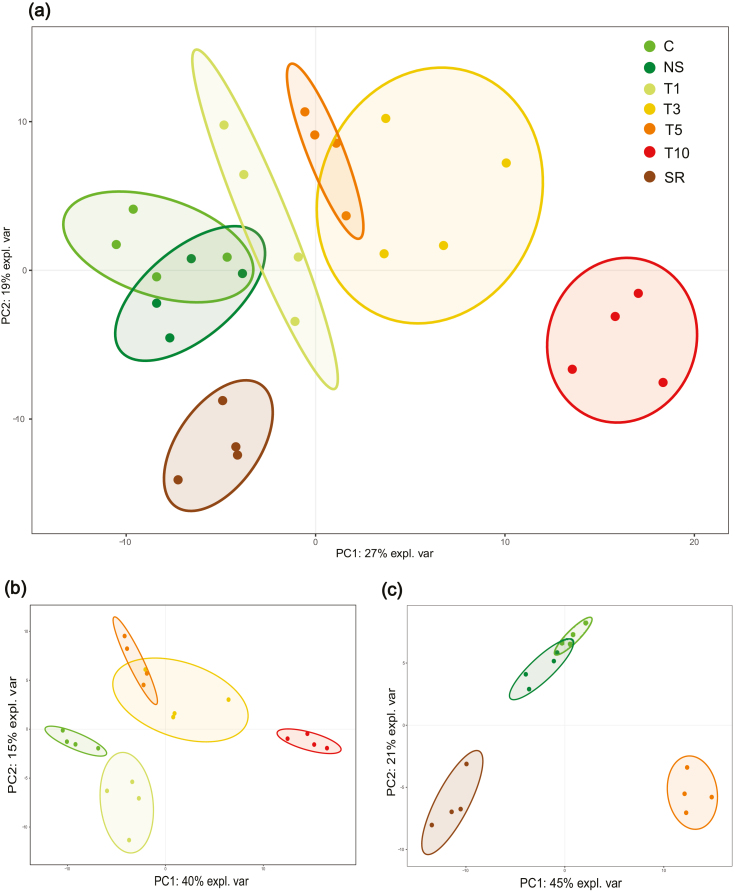
Principal component (PC) analysis of ANOVA-filtered (*q*-value <0.05) nuclear proteins of needles of *Pinus radiata* seedlings subjected to heat stress in Phase I. (a) All sample groups analysed together (C, T1, T3, T5, T10, SR, and NS); (b) heat-treated sample groups only analysed together (C, T1, T3, T5, T10); and (c) recovered samples groups only analysed together (C, T5, SR, and NS). Ellipses indicate a 0.90 confidence level. The sampling times correspond to the experimental set-up shown in Fig. 1.

In order to reduce the complexity of the results, multivariate analyses including PCA and *K*-means were performed. PCA showed that the first two components accounted for 46% of the total variance (Fig. 4a). The treatments were separated into three mains groups: control plants (C and NS), heat-stressed plants (T1, T3, T5, and T10), and recovered plants (SR). The variance gathered for each component was explained by analysing the proteins exhibiting the highest and lowest loadings for each component. Component 1 (PC1) seemed to be related to the heat-stress response (Table 1); thus, among the proteins in PC1 were heat-shock proteins (HSPs), proteins that have previously been related to cold stress such as LOS4 (Gong *et al.*, 2004) and phosphoglycerate kinase (Bae *et al.*, 2003; Escandón *et al.*, 2017), and elements involved in proteome and transcriptome reorganization such as the arginine/serine-rich splicing factor (SRSF) and the 40S ribosomal protein S30.

**Table 1.** Top-scoring protein loadings of PCA using all treatments in the analysis

Accession	Description	PC1	Accession	Description	PC2
Contig_08474_6_2	40S ribosomal protein S20-2	0.09984011	PITA_000011495-RA	Importin-5	0.08923564
Contig_08057_5_3	Temperature-induced lipocalin-1	0.09884283	Contig_16577_4_2	Eukaryotic translation initiation factor 4B1	0.08897829
Contig_04845_4_4	60S ribosomal protein L23a	0.09844721	Contig_02884_6_10	Nucleic acid-binding, OB–fold	0.08623538
Contig_03501_4_2	Heat-shock protein SSA1	0.09800322	Contig_20257_6_3	Putative mitochondrial ribosomal protein S1	0.07609981
PITA_000008167-RA	Phosphoglycerate kinase	0.09760940	Contig_09049_5_2	Eukaryotic initiation factor 4A	0.07514372
Contig_27779_5_7	40S ribosomal protein S30	0.09662240	Contig_02391_5_6	T-complex protein 1 subunit zeta 1	0.07285901
Contig_08531_6_3	FIB4	0.09484834	Contig_25893_4_3	Ras-related protein Rab-14	0.07086381
Contig_24839_5_5	DnaJ protein ERDJ3A	0.09462240	Contig_25697_4_2	Protein PLASTID TRANSCRIPTIONALLY ACTIVE 14	0.06774473
Contig_05091_5_3	RNA-binding protein 8A	0.09358250	Contig_01307_5_8	RuvB-like 2	0.05886918
Contig_08803_6_7	Small heat-shock protein	0.09339187	Contig_16521_5_5	Probable mediator of RNA polymerase II transcription subunit 37b	0.05774368
Contig_03893_5_1	Sm-like protein LSM3A	0.09323759	Contig_00337_4_3	Eukaryotic peptide chain release factor subunit 1	0.05671423
Contig_10255_6_9	Ribosomal protein L34	0.09225476	Contig_24539_6_4	Proline synthase co-transcribed bacterial homolog protein	0.05430940
Contig_08100_5_5	17.5 kd heat-shock family protein	0.09197843	Contig_03989_5_4	Probable prolyl 4-hydroxylase 4	0.05220210
Contig_29238_6_8	LOS4	0.09163144	PITA_000009545-RA	DEAD-box ATP-dependent RNA helicase 52B	0.05131146
Contig_05685_5_3	Flavin oxidoreductase	–0.09142443	Contig_20183_5_3	60S ribosomal protein L21	–0.12353489
Contig_08429_5_10	ATP-citrate (pro-S-)-lyase	–0.08595130	Contig_24269_4_3	NEDD8-activating enzyme E1 catalytic subunit	–0.12006481
Contig_09823_4_5	26S proteasome non-ATPase regulatory subunit 11	–0.08451837	PITA_000013247-RA	Phosphoenolpyruvate carboxylase	–0.11525289
Contig_08703_4_1	Coumarate 3-hydroxylase	–0.08385169	PITA_000049403-RA	Calcium-transporting ATPase	–0.11279927
Contig_20161_5_1	Protein plastid transcriptionally active 16, chloroplastic	–0.08058523	Contig_59142_6_5	Dolichyl-diphosphooligosaccharide-protein glycosyltransferase subunit STT3A	–0.10961290
Contig_59320_4_6	Delta(24)-sterol reductase	–0.07604593	Contig_20494_5_5	Sucrose-phosphatase 1	–0.10854364
Contig_25158_4_7	Regulator of transcription that contains myb domains	–0.07503385	Contig_02235_6_6	Sterol C7 reductase	–0.10680418
Contig_25158_4_7	Regulator of transcription that contains myb domains	–0.07503385	PITA_000069487-RA	Cullin-1	–0.10603460
Contig_20184_5_1	PPIase	–0.07490530	Contig_72397_4_2	Coatomer subunit epsilon	–0.10533729
Contig_04858_6_6	26S proteasome non-ATPase regulatory subunit 12 homolog B	–0.07314704	Contig_02419_5_10	Alpha/beta-Hydrolases superfamily protein	–0.10459699
Contig_05299_5_4	Peroxisomal acyl-coenzyme A oxidase 1	–0.07141400	Contig_08676_6_13	ERBB-3 BINDING PROTEIN 1	–0.10288798
PITA_000019791-RA	Arginine/serine-rich splicing factor RS34	–0.07138290	Contig_66792_6_4	LIM transcription factor homolog	–0.10176071
Contig_16059_6_5	Magnesium-protoporphyrin IX monomethyl ester [oxidative] cyclase	–0.0704459	Contig_04165_6_3	Signal recognition particle 54 kDa protein, chloroplastic	–0.10166426
Contig_65484_6_2	DNA topoisomerase 6 subunit B	–0.07037945	Contig_08771_5_6	Ran-binding protein 1 homolog b	–0.10163468

In the case of PC2, the biological interpretation remained unclear as it included proteins involved in a wide range of processes (signalization, ER transport, and DNA damage among others) (Table 1). This was probably due to an excess of variability as a consequence of mixing stress-recovery (SR) and stress response (T1, T3, T5, T10) processes in the same multivariate analysis. Separating the treatments into two major categories, namely heat-treated (HT) consisting of C, T1, T3, T5, and T10, and recovery (R) consisting of C, T5, SR and NS, caused the explained variance to increase to 55% and 67%, respectively. Nevertheless, the PC2 negative loadings for all the treatments (Table 1) may have constituted a cluster that is mainly composed of recovery and heat-stress memory proteins. Neddylation, a post-transcriptional modification directly linked to histone H2A, seemed to be occurring, since the NEDD8-activating enzyme E1 catalytic subunit was over-accumulated, concomitantly with histone H2A replenishment above basal levels. NEDD8 is covalently conjugated to H2A, and neddylation of H2A antagonizes its ubiquitylation (Li *et al.*, 2014).

PC analysis of HT plants and their controls indicated that PC1 classified the samples into those consisting of no stress (C), first and mid-term responses (T1, T3, and T5), and a long-term response (T10) (40% of explained variance; Fig. 4b), while PC2 explained the variation among the mid-term stress treatments. Analysing the proteins with the top-scoring loadings in PC1 (Table 2, Supplementary Table S3) identified small HSPs (sHSPs) and also proteins related to epigenetic and alternative-splicing regulation. Adenosylhomocysteinase (SAHH) and its product–competitive inhibitor S-adenosylmethionine (SAM) synthase, proteins involved in DNA and histone methylation, and the SM-like protein LSM3A (LSM3A), which is directly linked to alternative-splicing regulation, all play critical roles in the regulation of development-related gene expression (Perea-Resa *et al.*, 2012). Furthermore, two transcription factors belonging to the NF-Y family involved in histone methylation markers (Donati *et al.*, 2008) were found to be up-regulated in response to heat stress. These results suggested a fundamental change in cell organization leading to a new proteome profile. In addition, fatty acid metabolism and flavonoid biosynthesis also seemed to be altered, as indicated by changes found in PC1 to the proteins of the first steps of the phenylpropanoid biosynthesis pathway (coumarate 3-hydrolase, C3H) and PC2 (caffeic acid O-methyltransferase, COMT). Interestingly, in PC2, which separated the first response (T1) and the mid-term response (T3 and T5), a cluster of spliceosome-related proteins including heterogeneous nuclear ribonucleoprotein U-like protein 1 (HNRNP U-like1) and SRSF was found to be up-regulated proportionally to the exposure to heat stress (Table 2; PC2 positive loadings).

**Table 2.** Top-scoring protein loadings of PCA using heat-treated groups in the analysis

Accession	Description	PC1	Accession	Description	PC2
PITA_000010241-RA	Hap3/NF-YB transcription factor	0.10849829	Contig_10073_5_4	WD repeat-containing protein 5	0.14264195
Contig_08349_4_4	Eukaryotic translation initiation factor 3 subunit G	0.108405573	Contig_65355_4_6	Heme oxygenase 1, chloroplastic	0.12470135
Contig_08474_6_2	40S ribosomal protein S20-2	0.10807525	Contig_04940_5_3	Mitochondrial-processing peptidase subunit beta	0.11445940
Contig_00146_6_4	60S ribosomal protein L23	0.10679511	Contig_16521_5_5	Probable mediator of RNA polymerase II transcription subunit 37b	0.10870573
Contig_20076_6_9	Small heat-shock protein	0.10599791	PITA_000029470-RA	Arginine/serine-rich splicing factor	0.10703492
Contig_08747_5_4	Low molecular weight heat shock protein	0.10373032	Contig_08508_5_6	DNA/RNA-binding protein Alba-like protein	0.10095172
Contig_01261_5_1	Not annotated	0.10354882	Contig_04501_5_5	Trans-2,3-enoyl-CoA reductase	0.10088246
Contig_03893_5_1	Sm-like protein LSM3A	0.10322270	Contig_08798_5_8	Heterogeneous nuclear ribonucleoprotein U-like protein 1	0.09491046
Contig_24570_4_7	Alba DNA/RNA-binding protein	0.10280051	Contig_06439_5_1	Aldehyde dehydrogenase family 3 member F1	0.09329713
Contig_08913_5_6	17.6 kDa class II heat-shock protein	0.10254074	Contig_06458_4_1	DEAD-box ATP-dependent RNA helicase 53	0.08816753
Contig_04845_4_4	60S ribosomal protein L23a	0.10232330	PITA_000013006-RA	Protein Fes1A	0.08377962
PITA_000067325-RA	Hap3/NF-YB transcription factor	0.10180439	Contig_08477_6_7	Protein HEAT-STRESS-ASSOCIATED 32	0.08170930
PITA_000008614-RA	Heterogeneous nuclear ribonucleoprotein 27C	0.10144183	Contig_03427_6_1	Novel plant SNARE 13	0.08165179
Contig_00369_6_3	22.0 kDa class IV heat-shock protein	0.10101350	Contig_08351_6_5	Pre-mRNA cleavage factor Im 25 kDa subunit 2	0.07849228
Contig_65484_6_2	DNA topoisomerase 6 subunit B	–0.04800358	Contig_24087_5_5	40S ribosomal protein S10-3	–0.13122584
Contig_04359_5_7	Salt tolerance protein 1	–0.04948116	Contig_25619_6_7	Dehydrogenase/reductase SDR family member 4	–0.13148747
Contig_04026_5_7	Thiamine thiazole synthase	–0.05180065	Contig_05639_5_6	EF1Bgamma class glutathione S-transferase	–0.13215248
Contig_59320_4_6	Delta(24)-sterol reductase	–0.05808409	PITA_000024378-RA	Ribosomal protein	–0.13488120
Contig_00925_5_2	S-adenosylmethionine synthase	–0.05863694	PITA_000013091-RA	Enoyl-CoA hydratase/ 3-hydroxyacyl-CoA dehydrogenase	–0.13631146
Contig_20653_6_3	Beta-glucosidase 42	–0.06311270	Contig_65714_6_2	DNA damage-inducible protein 1	–0.13736195
Contig_20490_5_9	Peroxisomal fatty acid beta-oxidation multifunctional protein	–0.06784455	Contig_00467_5_1	Chalcone synthase	–0.13942408
Contig_47125_5_1	UDP-glucose 6-dehydrogenase 3	–0.07109042	PITA_000050783-RA	Caffeic acid O-methyltransferase	–0.14072545
Contig_20550_4_3	S-adenosylmethionine synthase 5	–0.07191758	Contig_08520_6_3	Male gametophyte defective 1	–0.14072718
Contig_20161_5_1	Protein plastid transcriptionally active 16, chloroplastic	–0.07216767	Contig_08743_4_5	Protein disulfide-isomerase	–0.14128362
PITA_000026132-RA	Calcium-dependent phosphotriesterase superfamily protein	–0.07428447	Contig_04879_4_8	ATP synthase subunit D	–0.14943307
Contig_05299_5_4	Peroxisomal acyl-coenzyme A oxidase 1	–0.074663742	Contig_17049_6_1	Aldehyde dehydrogenase	–0.15208964
Contig_08703_4_1	Coumarate 3-hydroxylase	–0.07761407	Contig_50733_6_1	Histone H2B	–0.15360951
PITA_000014101-RA	Adenosylhomocysteinase	–0.08187826	Contig_08344_4_2	Fructose-bisphosphate aldolase	–0.15390372
Contig_09823_4_5	26S proteasome non-ATPase regulatory subunit 11	–0.08249182	Contig_24598_5_2	Ubiquitin carboxyl-terminal hydrolase 6	–0.15449423

PC analysis of the recovery treatments (Fig. 4c) showed the nuclear proteins that did not return to the basal level after a 5-d heat treatment even after a month in control conditions. PC1 (45% of explained variance) distinguished stress-induced proteome changes related to adaptation, with H2A and H2B (negative loadings; Table 3) being markers of acquired memory (Kumar and Wigge, 2010; Liu *et al.*, 2015), and spliceosome-related proteins such as HNRNP U-like1 and HNRNP 27C (positive loadings) being markers of heat exposure. PC2 (21% of explained variance) showed stable induced markers in the modulation of the proteome profile, confirming the importance of molecular chaperones (sHSP and PPIase), ER–chloroplast crosstalk (e.g. UDP-sulfoquinovose synthase) (Higashi *et al.*, 2015), and DNA mismatch repair processes (HPMS5 homologue) (Horii *et al.*, 1994).

**Table 3.** Top-scoring protein loadings of PCA using the recovered groups in the analysis

Accession	Description	PC1	Accession	Description	PC2
Contig_20554_5_5	RNA-binding (RRM/RBD/RNP motifs) family protein	0.09725004	Contig_04359_5_7	Salt tolerance protein 1	0.10582088
Contig_25049_5_1	60S ribosomal protein L15-1	0.09537084	A0A0A7REG5	Late-embryogenesis abundant protein LEA7-1	0.10036371
Contig_00952_4_5	Glycine-rich RNA-binding protein RZ1B	0.09362835	Contig_59320_4_6	Delta(24)-sterol reductase	0.09246605
Contig_08508_5_6	DNA/RNA-binding protein Alba-like protein	0.08874953	Contig_06027_5_11	Casein kinase II subunit alpha	0.08700034
Contig_24839_5_5	DnaJ protein ERDJ3A	0.08840664	PITA_000052104-RA	WD40-like transcription factor	0.08656163
Contig_20209_5_2	Reticulon-like protein B2	0.08611899	Contig_20324_4_3	Glutamate decarboxylase	0.08429516
PITA_000008614-RA	Heterogeneous nuclear ribonucleoprotein 27C	0.08574246	PITA_000028702-RA	Not annotated	0.07696673
Contig_20357_5_7	SNF2 transcription factor	0.08557046	Contig_65484_6_2	DNA topoisomerase 6 subunit B	0.07567501
Contig_16279_4_8	Heat-shock 90/70 organizing protein	0.08540847	PITA_000026132-RA	Calcium-dependent phosphotriesterase superfamily protein	0.07303465
Contig_04580_4_4	Heterogeneous nuclear ribonucleoprotein 27C	0.08540827	Contig_25619_6_7	Dehydrogenase/reductase SDR family member 4	0.07210680
Contig_01307_5_8	RuvB-like 2	0.08530294	PITA_000008806-RA	CSC1-like protein ERD4	0.06854720
Contig_24776_4_10	Peroxisomal targeting signal 2 receptor	0.08507687	Contig_17049_6_1	Aldehyde dehydrogenase	0.06160797
Contig_08798_5_8	Heterogeneous nuclear ribonucleoprotein U-like protein 1	0.08487486	Contig_08520_6_3	Male gametophyte defective 1	0.06156818
Contig_04651_6_4	Translocon-associated protein (TRAP), alpha subunit	0.08464990	Contig_02419_5_10	Alpha/beta-Hydrolases superfamily protein	0.06083877
Contig_04385_5_7	RS9, ribosomal protein 9	–0.08880446	Contig_04940_5_3	Mitochondrial-processing peptidase subunit beta	–0.11184213
Contig_20183_5_3	60S ribosomal protein L21	–0.08889407	Contig_64893_4_5	Serine-threonine kinase receptor-associated protein	–0.11201250
Contig_20232_6_4	40S ribosomal protein S15	–0.08903394	PITA_000076143-RA	Isoeugenol synthase 1	–0.11230293
Contig_24502_6_3	50S ribosomal protein L10, chloroplastic	–0.08950550	Contig_20257_6_3	Putative mitochondrial ribosomal protein S1	–0.11566757
PITA_000016128-RA	Transposon protein, putative, unclassified, expressed	–0.08954858	Contig_29238_6_8	LOS4	–0.11637011
Contig_20141_5_2	Cucumisin	–0.08971181	Contig_01514_4_20	Riboflavin synthase	–0.11840414
Contig_20568_6_6	60S ribosomal protein L38	–0.09020170	PITA_000029470-RA	Arginine/serine-rich splicing factor	–0.11957617
Contig_04656_5_3	NADH dehydrogenase [ubiquinone] 1 alpha subcomplex subunit 13-A	–0.09050891	PITA_000018322-RA	HPMS5 protein	–0.12056262
Contig_04066_5_5	Cinnamate 4-hydroxylase	–0.09274560	Contig_25015_4_23	AMPSase	–0.12100694
PITA_000045727-RA	60S ribosomal protein L26-1	–0.09390991	Contig_09499_6_9	T-complex protein 1 subunit zeta	–0.12318434
Contig_20653_6_3	Beta-glucosidase 42	–0.09450440	PITA_000000788-RA	Eukaryotic translation initiation factor 2 gamma subunit, putative	–0.12775447
Contig_16356_6_4	17.5 kd heat-shock family protein	–0.09517232	Contig_01318_6_6	AtSUFE	–0.13232372
Contig_27539_5_7	Histone H2A	–0.09627523	Contig_20216_6_1	PPIase	–0.14965785
Contig_50733_6_1	Histone H2B	–0.09696277	Contig_04150_5_9	UDP-sulfoquinovose synthase, chloroplastic	–0.15114027

### *K*-means clustering analysis of nuclear proteins and 5-mdC immunolocalization in needles identify a central role of the epigenome in heat-stress response

**Fig. 5.**
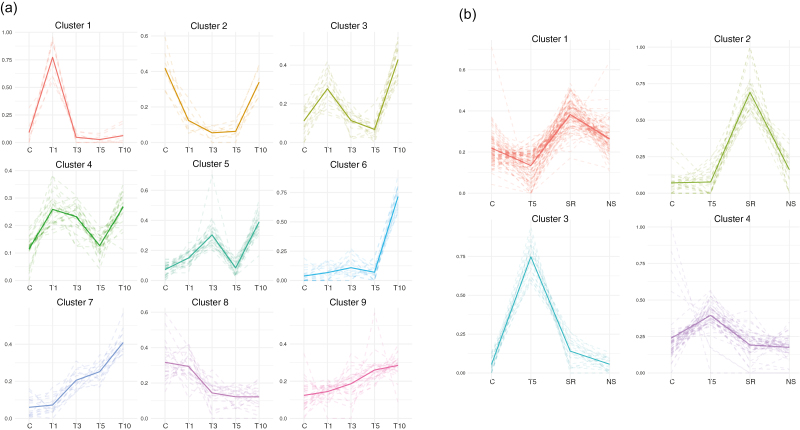
*K*-means clustering of differentially accumulated (*q*<0.05) nuclear proteins in needles of *Pinus radiata* seedlings subjected to heat stress. (a) Heat-treated groups (C, T1, T3, T5, T10) and (b) recovered groups (C, T5, SR and NS). The solid line shows the mean for each cluster and dashed lines show individual patterns. The sampling times correspond to the Phase I experimental set-up shown in Fig. 1. (This figure is available in colour at *JXB* online.)

Co-accumulation analysis in the heat-treated and recovery groups was investigated using *K*-means clustering analysis. Proteins of the heat-treated periods were clustered in nine groups (Fig. 5a). The elements involved in the first response to heat were grouped in cluster 1 and included the peak-and-run class heat-shock factor (HSF) A6b, while proteins related to stress signalling and responses such as calmodulin-like protein 3 (CML3), LSM3A, HSPs, and ribosomal proteins were found in cluster 6. Clusters 7 and 9 showed a pattern of continuous increases; these clusters included two of the most well-known proteins related to acquired thermotolerance, HSP101 and heat stress-associated 32 (HSA32) (Wu *et al.*, 2013). In contrast, cluster 8 showed a decreasing pattern; included in this cluster were proteins such as SAM synthase and SAHH, both key elements for epigenetic reorganization.

The nuclear proteomes of the recovery treatments were grouped into four different clusters (Fig. 5b). Clusters 1 and 2 showed a similar trend with a clear peak in SR, which indicated groups of proteins up-regulated 1month after the 5-d heat-stress treatment. While cluster 1 comprised histone H2A and some ribosomal proteins that were decreased at the stress point T5, cluster 2 showed over-accumulated proteins only in plants recovered from the stress. This cluster comprised key proteins including: SAM synthase that are strongly related to DNA, RNA, and histone methylation (Bender, 2004); calcium-binding protein CML13, a calcium sensor that is possibly involved in heat sensing and has previously been described as a cold-inducible nuclear protein in Arabidopsis (McCormack and Braam, 2003; Saidi *et al.*, 2011); Hap3/NF-YB transcription factors; NEDD8 E1, which is essential for DNA damage response modulation (Brown and Jackson, 2015); and histones H1 and H4. Cluster 4 included stress-related proteins (e.g. HSP101, FIB4, and LOS4, which were also identified in PCA HT loadings; Table 2) that returned to control levels when the heat exposure ended. Cluster 3 followed the same trend as the proteins related heat stress; however, in this group significant differences were detected between the levels in NS and SR plants, with a slight increase in SR protein abundance compared to the control NS (e.g. FES1A, HNRNP U-like 1, BAX inhibitor 1) (Supplementary Table S4).

**Fig. 6.**
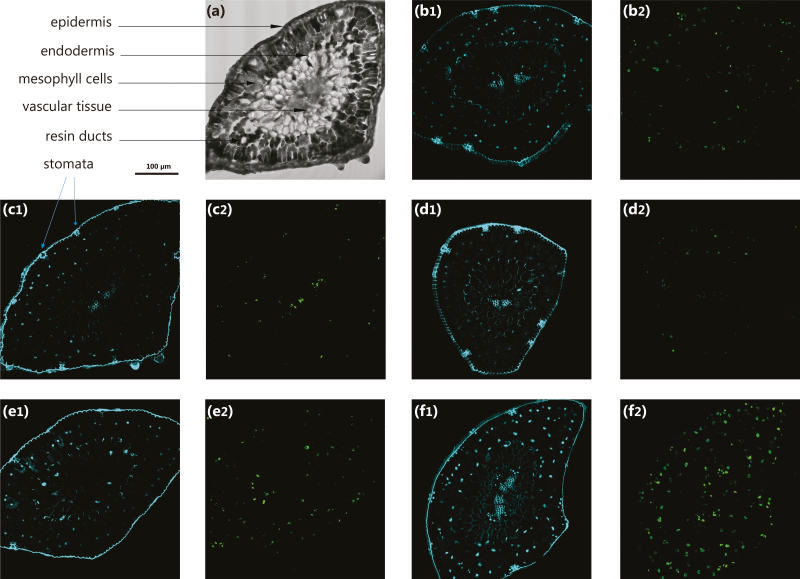
Immunolocalization using 5-methylcytosine (5-mC) in transverse sections of needles of *Pinus radiata* seedlings subjected to heat stress. Differential interference contrast images are shown in the first image of each pair, and the fluorescence signal is shown in the second. The sampling times correspond to the experimental set-up shown in Fig. 1: (a) control plants (C); (b) T1; (c) T3; (d) T5; (e) SR. (1) Nuclei marked with DAPI, and (2) with 5-mC. (This figure is available in colour at *JXB* online.)

Epigenetic reorganization related to adaptation was further examined by immunolocalization analysis of 5-mdC in needles collected during the first phase of the experiment (Fig. 6; the negative control is shown in Supplementary Fig. S3). The 5-mdC signal was decreased from the initial control to T3 in the cell nucleus, with the scarce signal found in stressed plants at T1 limited to the vascular tissue, in accordance with the SAM synthase accumulation profile found by the *K*-means analysis. In T5, the signal started to increase, reaching the highest levels of DNA methylation in stress-recovered (SR) plants.

### sPLS analysis reveals a complex network of nuclear protein interactions involved in the heat-stress response

**Fig. 7.**
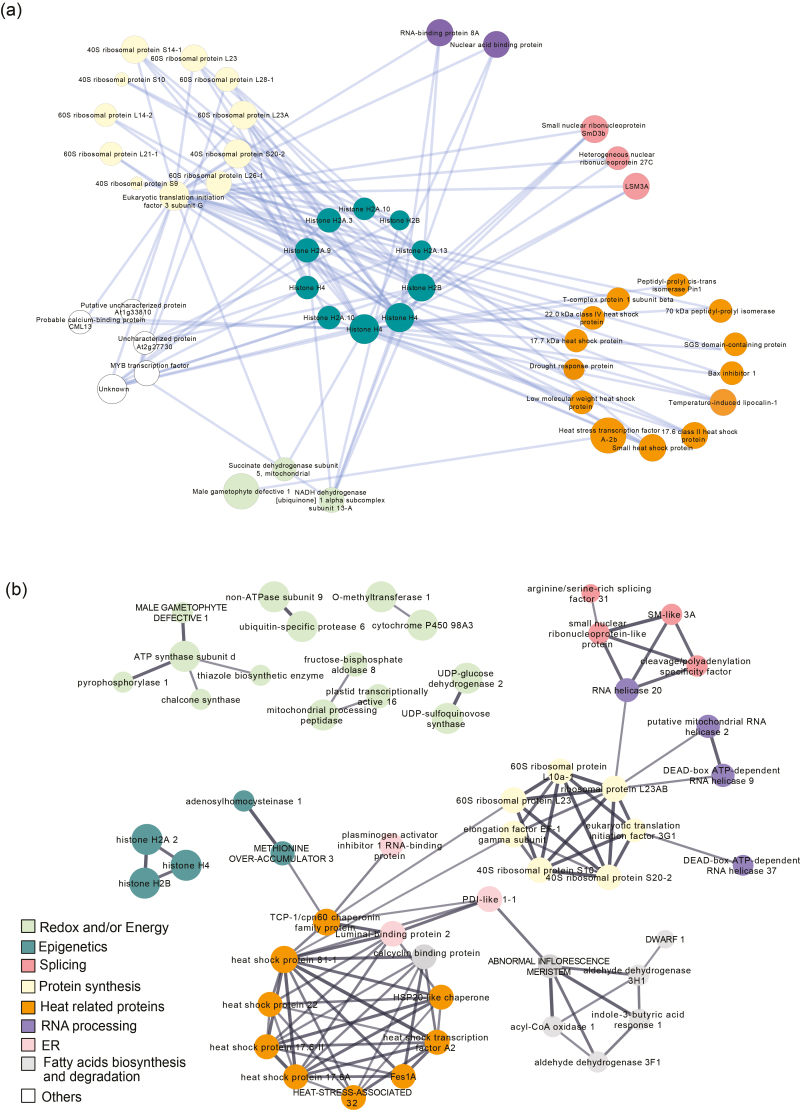
Integrative analysis of nuclear proteins involved in heat stress and thermopriming process in needles of *Pinus radiata* seedlings. (a) sPLS-based network built using transcription factors (TFs) and regulators identified with the TF predictor tool as the predictor matrix for changes in the rest of nuclear proteome. The correlation cut-off was 0.75, and the node size was calculated accordingly to radiality. (b) STRING-based network of the 40 most-relevant proteins (20 top-scoring positive loadings and 20 top-scoring negative loadings) in PCA components 1 and 2. Selected proteins were blasted against the STRING database of the model species *Arabidopsis thaliana* and those with a minimum of homology of 60% were employed to build the network. Network edges indicate a biological correlation at least of 0.7 from experimental or curated database resources.

Sparse partial least-square (sPLS) analysis produced networks that were constructed by considering transcription factors and regulators as the predictor matrix for the rest of the differentially accumulated nuclear proteins in heat-treated (HT) groups (Fig. 7a, Supplementary Table S5A). This pinpointed the importance of sHSPs, ribosomal proteins, and spliceosome-related proteins during heat stress (e.g. LSM3A and the small nuclear ribonucleoprotein SmD3b). Histones (H2A, H2B, and H4) and eukaryotic translation initiation factor 3G (eIF3G) were shown to play major roles in managing the heat-stress response, being the nodes selected according to the 0.75 threshold to explain the relations among the nuclear proteins.

The DnaJ ERDJ3A protein (a molecular chaperone) and glycine-rich protein were the unique nodes negatively related to net CO_2_ assimilation rate and transpiration (Supplementary Fig. S4, Supplementary Table S5B) in an sPLS-based network where proteins predicted leaf gas-exchange parameters. Coumarate 3 hydroxylase, coumarate 4 hydroxylase, and DNA topoisomerases 6A and B were down-regulated in heat-treated plants and were positively related to the photosynthesis parameters shown in the network.

Additional biological functional analysis of protein–protein interactions was performed using the STRING (Szklarczyk *et al.*, 2017) and Arabidopsis databases. Proteins selected using the STRING database from the 20 highest positive and negative loadings of the first two components in the PC analysis of heat-treated plants (Fig. 4b, Table S3) are shown in the network in Fig. 7b. Clear functional interactions were observed between the nuclear proteins identified, which put the biological clusters in line with the mathematical correlations established using sPLS analysis.

The functional interaction analysis showed clusters that were devoted to HSPs: ribosomal activity, epigenetics, fatty acid metabolism, and RNA processing and splicing. Under unfavourable environmental conditions, new protein biosynthesis is necessary for proteosomal rearrangement in response (Escandón *et al.*, 2017; Pascual *et al.*, 2017). This was reflected in the connection between spliceosome proteins and the ribosomal machinery, and between them and HSPs. In addition, HSPs were directly connected to epigenetic regulation (SAM synthase and SAHH), showing the relevance of quick regulation of HSP expression. Epigenetic proteins showed the highest values at T3 and T5, key points related to the acclimation process and adaptation, respectively (Supplementary Fig. S5). An independent cluster related to redox, flavonoid biosynthesis, and energy processes was also found.

### Targeted transcriptome analysis and physiological measurements support the better performance of primed plants upon a second round of stress

**Fig. 8.**
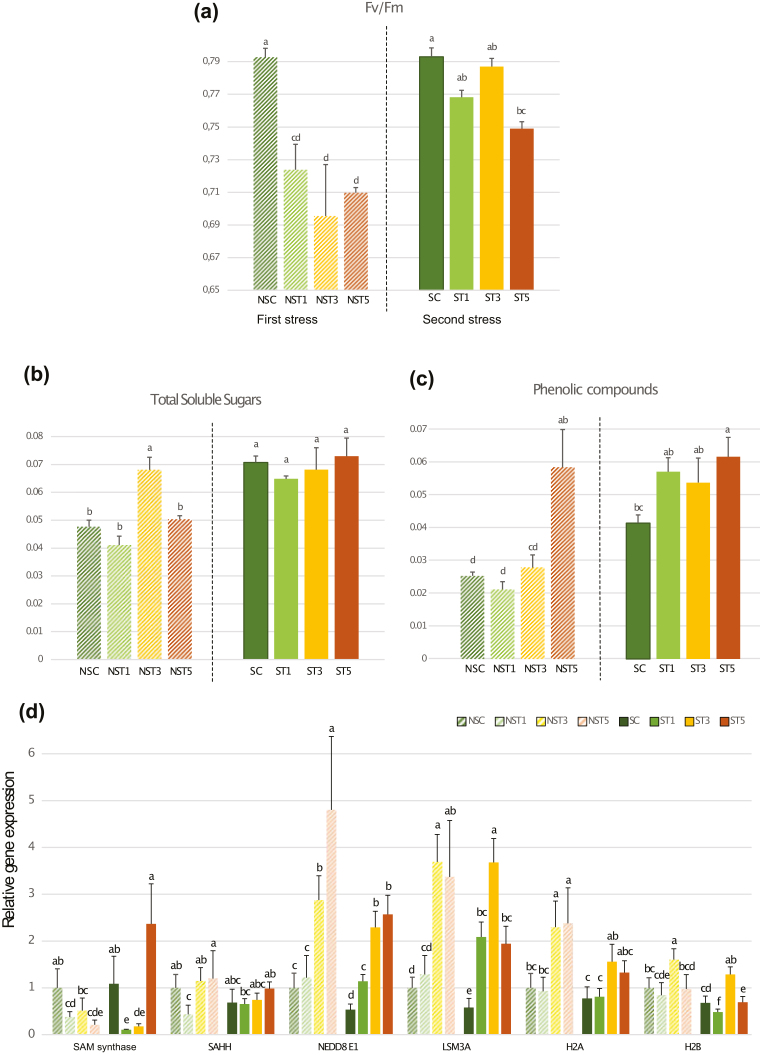
Physiological and gene expression effects observed in needles of *Pinus radiata* seedlings subjected to heat stress. (a) Maximum yield of photosystem II, (*F*_v_/*F*_m_), (b) content of total soluble sugars (TSS), (c) content of phenolic compounds, and (d) relative expression of candidate genes selected according to multivariate and integrative analysis. All measurements were taken on heat-primed and non-primed plants of experimental Phase II, as detailed in Fig. 1. Data are means (±SE), *n*=4. Different letters indicate significant differences as determined by ANOVA followed by Tukey’ HSD test differences (*P*<0.05). (This figure is available in colour at *JXB* online.)

To further validate the potential acquisition of memory after exposure to stress, we analysed the physiological performance and gene expression of nuclear candidate biomarkers in heat-primed and non-primed plants (experimental Phase II, Set I and Set II plants; Fig. 1). Primed and non-primed plants showed significant differences in their performance when exposed to the stress (Supplementary Fig. S6. The plants that were being subjected to their first exposure to stress suffered more photosynthetic damage in comparison with thermoprimed plants (Fig. 8a). The contents of total soluble sugars and total phenolics were significantly higher in primed plants (Fig. 8b, c), which indicates a physiological preconditioning to stress (Jesus *et al.*, 2015). These results therefore provided indications of a stable acquired thermotolerance.

The molecular responses were further examined by analysing the expression levels of genes coding for some of the key proteins previously identified in the multivariate analyses, covering DNA methylation (*SAM synthase* and *SAHH*), splicing (*LSM3A*), DNA repair (*NEDD8 E1*). and nucleosome assembly (*H2A* and *H2B*) (Fig. 8d). Primed (second stress) and non-primed (first stress) plants showed the same gene expression profiles across the heat exposure time-points, with the exception of *SAM synthase*. However, higher expression values were found in first-stressed plants for most of the genes and times. *SAM synthase* showed a particular behaviour required for DNA methylation, and exhibited control-like expression in the ST5 treatment in thermoprimed plants, while in first-stressed plants its levels were consistently low.

The better performance of thermoprimed plants with regards to photosynthetic activity coupled to the lower expression of candidate genes and the recovery of methyl cycle enzyme expression during the stress all support the hypothesis of stable acquired thermotolerance guided by epigenetic events.

## Discussion

### Integrative analysis of the nuclear proteome confirms proteomic rearrangement and small HSPs as essential mechanisms to maintain plant function after initial heat stress

High temperature has a great impact on plant physiology (Escandón *et al.*, 2017), and many and complex processes are involved in heat-response signalling. Recent studies in plants have elucidated the complex transcriptional regulatory networks involved in high-temperature responses (Ohama *et al.*, 2017; Ling *et al.*, 2018). Our study was focused on studying the nuclear proteome under heat stress; the nucleus plays an essential role during genome organization, different phases of cellular development, and physiological responsiveness through regulated gene expression. Thus, identification of nuclear proteins represents an initial step towards gaining new insights into cell responses to heat stress in *P. radiata*. The nuclear proteome is highly dynamic, changing its composition in response to environmental and intracellular stimuli (Pascual *et al.*, 2016) in order to guide the subsequent remodulation of the global proteome (Narula *et al.*, 2013), and it provides useful information about the mechanisms underlying the heat-stress adaptation processes in *P. radiata* at a transcriptional level.

In addition to HSPs, our study identified different regulation steps involved in epigenomic-driven gene regulation, several transcription factor families, and a variety of RNA-associated functions (spliceosome, proteasome, and mRNA surveillance). The results of all these changes were subsequently detectable in the over-accumulation of the translasome machinery (ribosomal proteins and eukaryotic initiation factors) needed to carry out the required cell reorganization. We also found differential responses to short-, mid-, and long-term heat exposure, as well as stable histone H2A-related heat-induced markers that were established during the recovery phase.

Photosynthetic activity was clearly impaired by heat stress in *P. radiata*, as observed from leaf gas-exchange parameters (Fig. 2) and heatmap clustering (Fig. 3). These results, in accordance with those of Buchner *et al.* (2015), showed that under heat stress photorespiration metabolism was favoured, and in the extreme treatment for 10 d it led to fermentation. We found differences in the gas-exchange parameters such as transpiration rate and stomatal conductance, with values for recovered plants exceeded those of the controls. Together with the alterations that we observed in the nuclear proteome, this provided a sign of epigenetic regulation; the biological processes were durable even in the absence of stress, indicating possible memory effects, which have already been described in other model species (Ling *et al.*, 2018).

Heatmapping revealed two groups associated with the stress treatments, namely those related to mid- (T3 and T5) and long-term (T10) exposure, and those related to short-term (T1), control, and recovery treatments, which emphasized the wide differences between stress and recovery events. Since the T10 treatment severely damaged the plants, the T5 treatment was used for priming of the plants carried forward to Phase II of the experiment (Fig. 1). A recent study by Ling *et al.* (2018) refers to thermopriming as an event of non-lethal exposure to heat stress that allow plants to survive subsequent and otherwise lethal conditions.

Using multivariate analyses including PCA and *K*-means, together with integrative approaches and a comprehensive analysis of the generated sPLS networks with biological correlations and the STRING tool, we were able to uncover several links between key proteins in relation to both the stress and memory responses. As expected, we found high abundances of HSPs, ER molecular chaperones (DnaJ), and ribosomal machinery, as previously reported by Escandón *et al.* (2017). Several relevant players and pathways were identified that have also previously been described in heat and cold responses, such as LOS4 (Gong *et al.*, 2002, 2004), phosphoglycerate kinase (Escandón *et al.*, 2017), and polyphenol biosynthesis through the alteration of COMT. These results not only validate the nuclear integrative approach used but also represent new findings, as discussed below.

### Stress-response and memory effects in conjunction with differential and opposite epigenetic patterns involving hypo- and hypermethylation

DNA methylation is a well-known epigenetic marker of transcriptional gene silencing, but it also occurs in the establishment of heterochromatin, transposon control, and genomic imprinting (Galindo-Gonzalez *et al.*, 2018). Two of the key enzymes regulating the methylation cycle, SAM synthase and SAHH, were identified as central elements in our integrative analysis of the nuclear proteome, with both decreasing proportionally with the stress exposure time (Fig. 5b, cluster 1). This seemed to indicate that heat stress drives hypomethylation, since S-adenosylmethionine (SAM) is a methyl-group donor and an essential methyltransferase co-factor, and SAHH is a methyl-cycle enzyme that is required for SAM regeneration and transcriptional gene silencing-mediated methylation. In addition, COMT, a precursor of fatty acid and flavonoid biosynthesis through phenylpropanoid biosynthesis, was found to be over-accumulated uniquely in the short-term response (T1) and this is also a SAM-dependent methyltransferase. Hence, contributing to the depletion of SAM and its consequences, the DNA hypomethylation observed in T1 and T3 (Fig. 6) could initially be the result of the overproduction of fatty acids required for the short-term response (Escandón *et al.*, 2017). In the mid- and long term, enzymes implicated with flavonoid synthesis are essential for successful adaptation and favour protein biosynthesis in the cell cytoplasm, which is of great importance at this point when the translasome machinery is clearly up-regulated and has a high demand for HSPs.

Interestingly, SAM synthase and SAHH were found to among the major differences between heat-stressed and stress-recovered plants (T5 versus SR); both were depleted by the stress, but higher levels were detected in the stress-recovered plants (Fig. 5b). Moreover, stress-induced demethylation has been found to relax chromatin structure, thereby allowing enhanced transcription and proteosomal rearrangement (Shilatifard, 2006; Santos *et al.*, 2011), which has been linked to heat-tolerant genotypes in other plant species (Gao *et al.*, 2014). DNA hypermethylation levels in the long-term treatments and in recovered plants (Fig. 6), whose adaptation to heat stress had started, also correlated with the high expression level of *SAM synthase* quantified in primed plants (Fig. 8b). This behaviour shows the pivotal role of epigenetics.

These results support the hypothesis that environmental factors (including temperature and other stresses) are almost certainly more important in changes in DNA methylation than in those that occur spontaneously or that have a genetic basis (Dubin *et al.*, 2015). Over recent years it has been proposed that, as sessile organisms that can persist in the same location for a long time, plants may be particularly likely to exploit DNA methylation for rapid adaptation to changing environments (Valledor *et al.*, 2012; Kawakatsu *et al.*, 2017; Carbó *et al.*, 2019).

### Nucleosome structure and spliceosome functioning are altered by heat stress in relation to the thermopriming process

Priming and the establishment of stress memory can help plants to survive a variety of abiotic stress conditions, including heat (Filippou *et al.*, 2012; Tanou *et al.*, 2012). The maintenance of acquired thermotolerance is crucial for successful priming and tolerance to subsequent exposure to otherwise lethal temperatures (Ling *et al.*, 2018). The rebound effect pattern of proteins that was observed in clusters 1 and 2 in the *K*-means analysis of the recovered groups (Fig. 5b) seemed to indicate a priming process, since the highest levels of some key proteins were increased in recovered plants, such as histones H2A and H2B, and NEDD8 E1. The decrease of histone abundance during the first round of stress could be related to the formation of hexasomes, as described by Shaytan *et al.* (2015). Hexasomes are stable nucleosomes lacking H2A–H2B dimers, usually caused by RNA polymerase II transcriptional activity and by DNA repair processes. Accordingly, RNA polymerase II kinetics are accelerated under heat exposure (Jonkers and Lis, 2015; Chen *et al.*, 2016) and some DNA mismatch repair proteins such as the HPMS5 homologue and NEDD8 E1 are over-accumulated. This hypothesis fits with the over-accumulation of some probable mediators of RNA polymerase II transcription proteins that were identified as the proteins in cluster 9 that were increased by stress (Fig. 5a, Supplementary Table S4).

In parallel with hypomethylation events, the loss of H2A–H2B dimers in the nucleosome was counteracted in the recovery step (1 month in control conditions), providing a sign of an epigenetic memory-related process. Interestingly, both histones (Fig. 5b) and DNA methylation patterns (Fig. 6) were altered in order to enhance DNA accessibility by chromatin relaxation under heat stress. While chromatin relaxation marked the nucleosome status during the stress, a more compact nucleosome structure was found to occur after recovery, when the plant metabolism was again focused on development and growth pathways. In addition, in the targeted transcriptome analysis, thermolabile histones were found to be up-regulated, providing an insight into the importance of nucleosome occupancy and DNA accessibility in the non-primed and primed responses (Fig. 8d). However, thermoprimed plants showed a more moderate increase of H2A and H2B expression during the second round of stress (Fig. 8d), confirming the essential role of nucleosome regulation in the priming process.

Spliceosome-related proteins were also significantly altered by heat stress, as shown by PCA, the sPLS networks, and by STRING tools (Figs 4, 7). Spliceosomal activity was impaired under stress (Table 2) and then the abundance of spliceosomal proteins was increased beyond basal levels in recovered plants (Fig. 5b, Supplementary Table S4); as a consequence, mRNA surveillance and proteasome activities such as ubiquitination were found to have a pivotal role in the STRING and sPLS networks, as a link between the spliceosome and translasome machinery. This could also lead to alternative or impaired splicing events in relation to the thermopriming process (Ling *et al.*, 2018). Changes in gene expression and alternative splicing in primed and non-primed plants have revealed alternative splicing functions as a novel component of heat shock memory in Arabidopsis (Ling *et al.*, 2018). We found similar results, with primed seedlings also showing higher levels of LSM3A expression and faster evolution of the heat-stress response (Fig. 8d).

### Conclusions

This study shows for first time the dynamics of the nuclear proteome related to the heat-stress response and the thermopriming process. The depth and complexity of this study in relation to the number of proteins identified and analyses performed allowed a detailed depiction of this process, and revealed several crucial families of proteins that are involved in different key regulation steps such as proteasome reorganization, RNA-associated functions, epigenomic-driven gene regulation, and specific transcription factors previously unconnected to heat stress. In addition, histone H2A, alternative splicing, and methyl-cycle enzymes seem to be directly linked to the induction of thermopriming, and the active remodelling of the transcriptome and proteome that triggers the crucial processes involved in high-temperature responses and adaptation. This newly discovered priming-induced epigenetic memory may represent a general feature of heat-stress responses in conifers, and it may facilitate the development of novel approaches to improving survival of pine trees under extreme heat stress in the current context of climate change.

## Supplementary data

Supplementary data are available at *JXB* online.

Fig. S1. Membrane damage of needles exposed to temperatures up to 50 °C.

Fig. S2. Comparison of nuclear extraction phases.

Fig. S3. Negative control of 5-mdC immunolocalization analysis.

Fig. S4. sPLS-based network combining leaf gas-exchange parameters with nuclear proteins.

Fig. S5. Heatmap clustering analysis of the most relevant categories of nuclear protein species depicted in the protein–protein interaction networks.

Fig. S6. Representative images of primed and non-primed seedlings after exposure to heat stress.

Table S1. List of primers used for RT-qPCR.

Table S2. Nuclear protein identification, quantification, annotation, and univariate analysis.

Table S3. PCA loadings.

Table S4. *K*-means clustering of ANOVA-filtered nuclear proteins.

Table S5. Spare partial least-squares regression of the transcription regulator network, and photosynthesis and nuclear proteins network.

erz524_suppl_supplementary_figures_S1-S6

erz524_suppl_supplementary_table_S1

erz524_suppl_supplementary_table_S2

erz524_suppl_supplementary_table_S3

erz524_suppl_supplementary_table_S4

erz524_suppl_supplementary_table_S5
