# Supplementary material for: Integrative analysis of the nuclear proteome in Pinus radiata reveals thermopriming coupled to epigenetic regulation
Source: J Exp Bot. 2019 Nov 29;71(6):2040–57. doi: 10.1093/jxb/erz524 (PMC7094079; doi:10.1093/jxb/erz524)
Supplement: erz524_suppl_supplementary_figures_S1-S6 [file erz524_suppl_supplementary_figures_s1-s6.pdf]

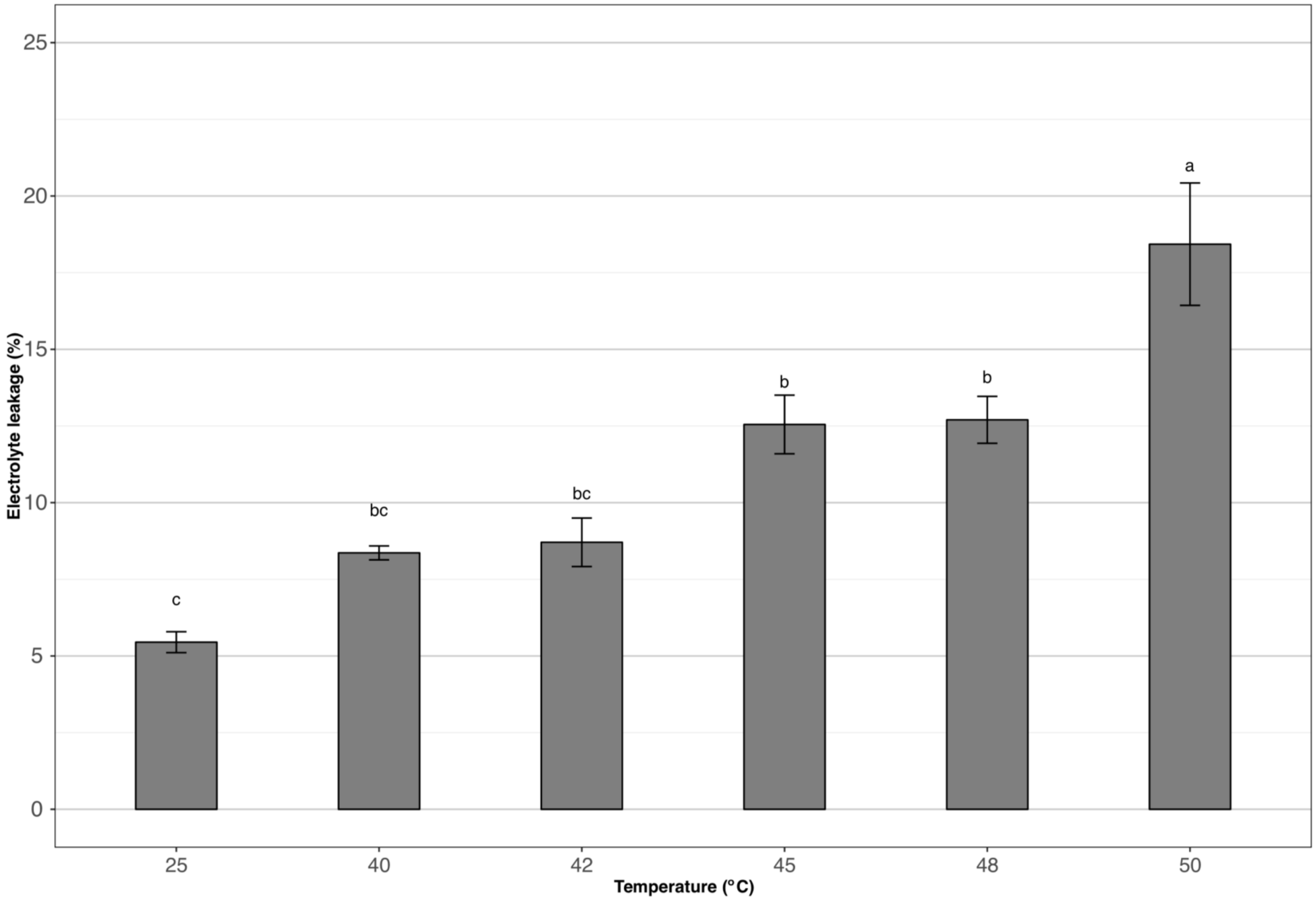

Figure S1: Percentage of membrane damage of *Pinus radiata* needles exposed to temperatures up to 50 ºC. Error bars represent standard deviation of three measurements. Treatments with the same letter are not significantly different.

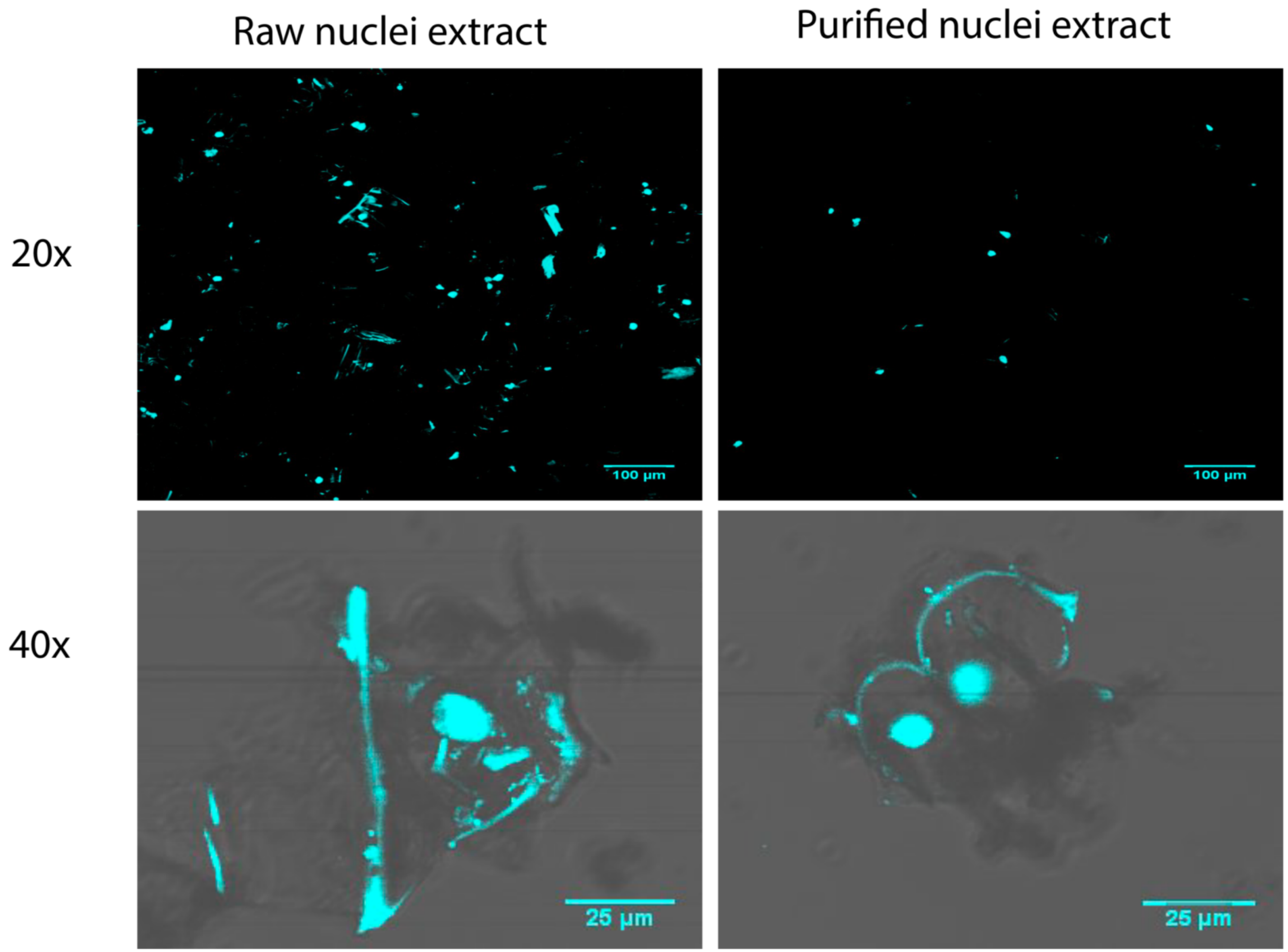

Figure S2: Comparison between nuclear extraction phases: a) raw nuclei (20X) and b) after purification (20X); c) Merged transmission and fluorescence image of raw nucleus (40X) and d) Merged transmission and fluorescence image of purified nuclei (40X)

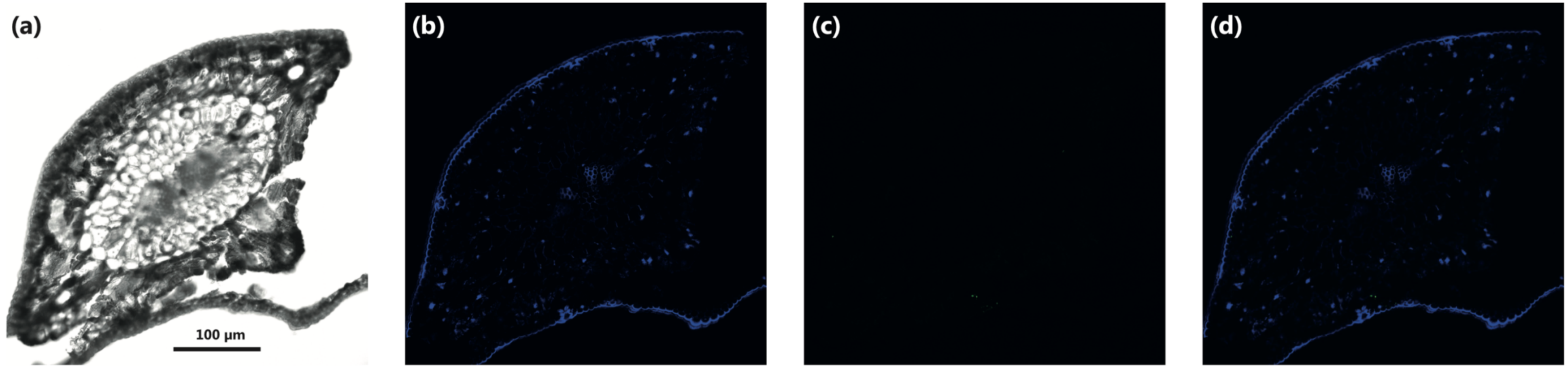

Figure S3: Negative control of 5-mC immunolocalization analysis. a) Differential interference contrast (DIC) in control plants; b) Blue signal of DAPI; c) Green signal of 5-mC monoclonal antibody and d) DAPI and 5-mC merged in transversal needle section.

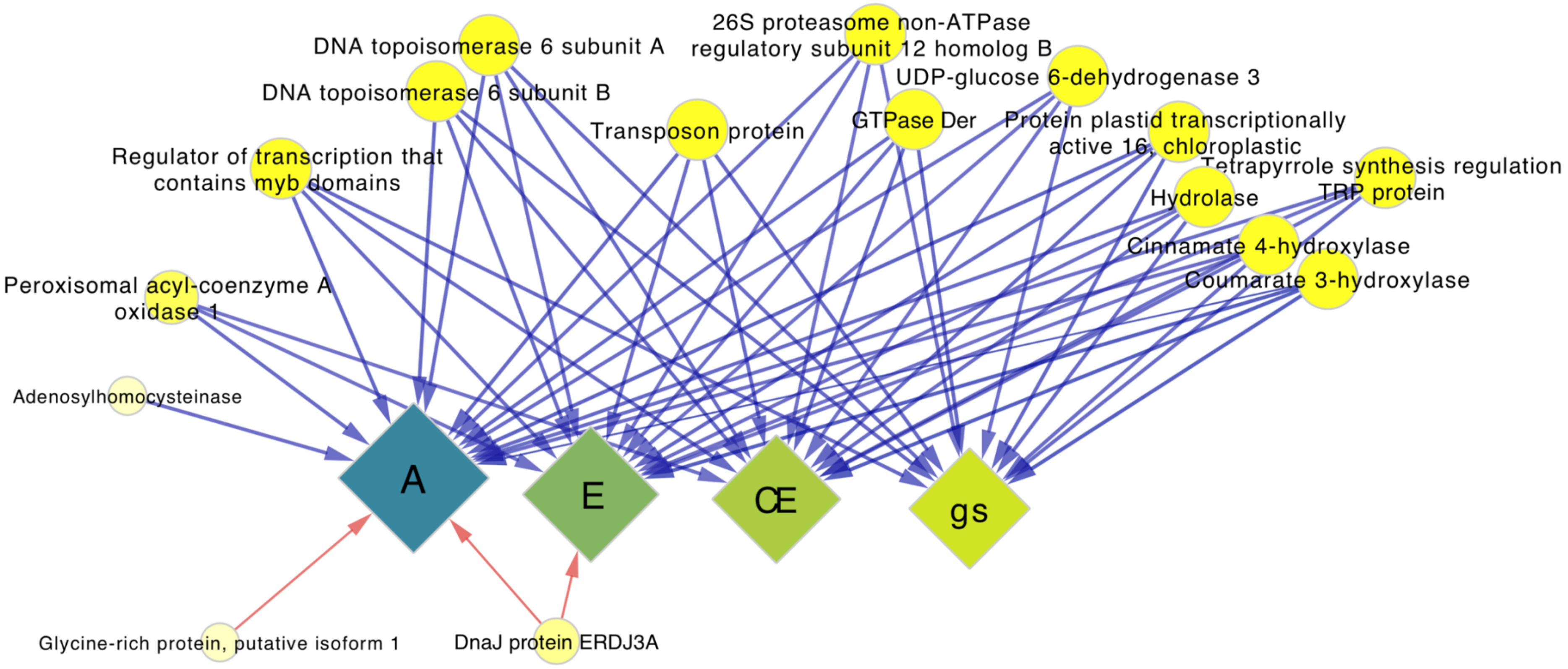

Figure S4: sPLS-based network combining leaf-gas Exchange parameters with nuclear proteins, positive correlations are denoted in blue and negatives in red

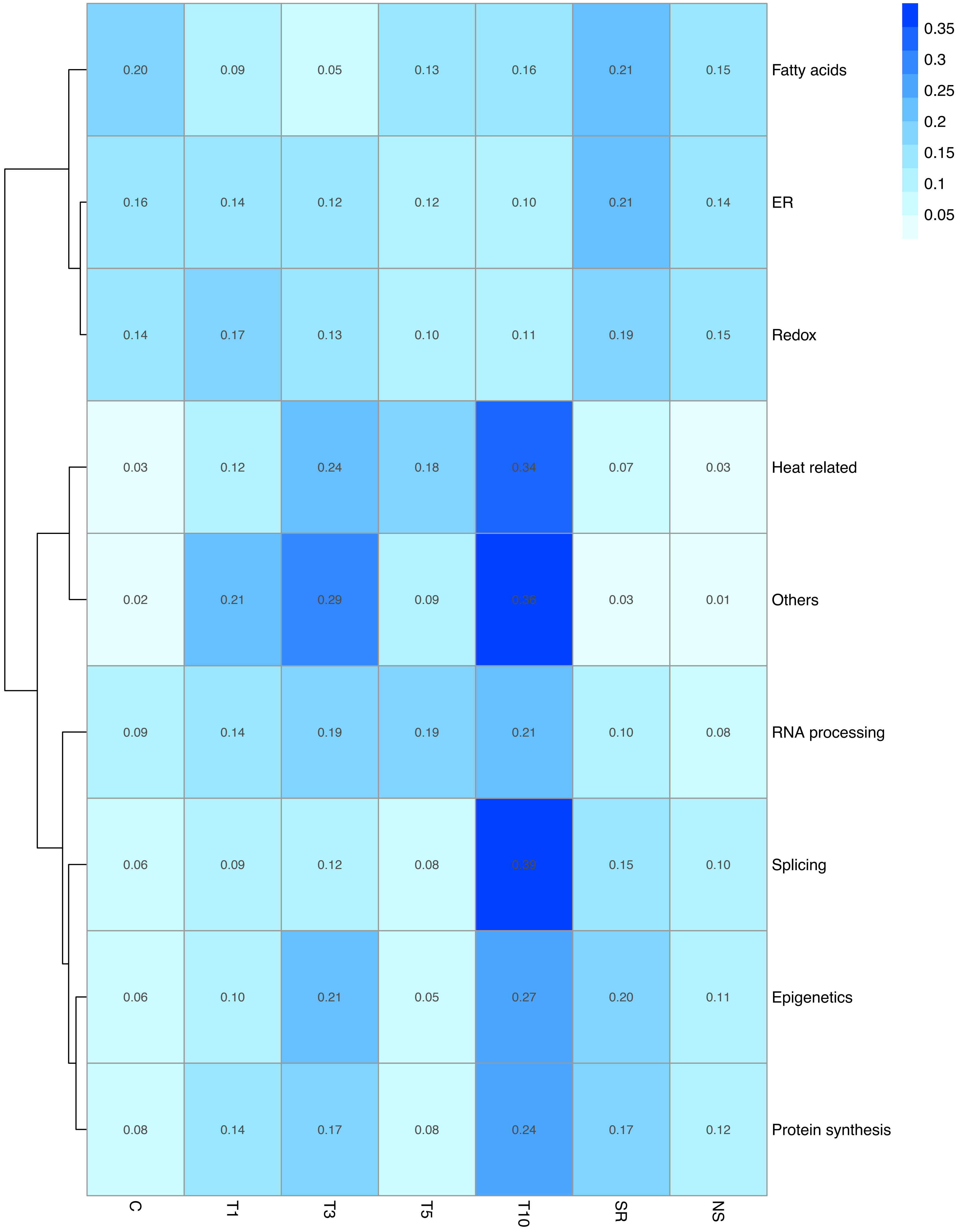

Figure S5: Heatmap-Clustering analysis of most relevant categories of nuclear proteins species depicted in protein-protein interaction networks

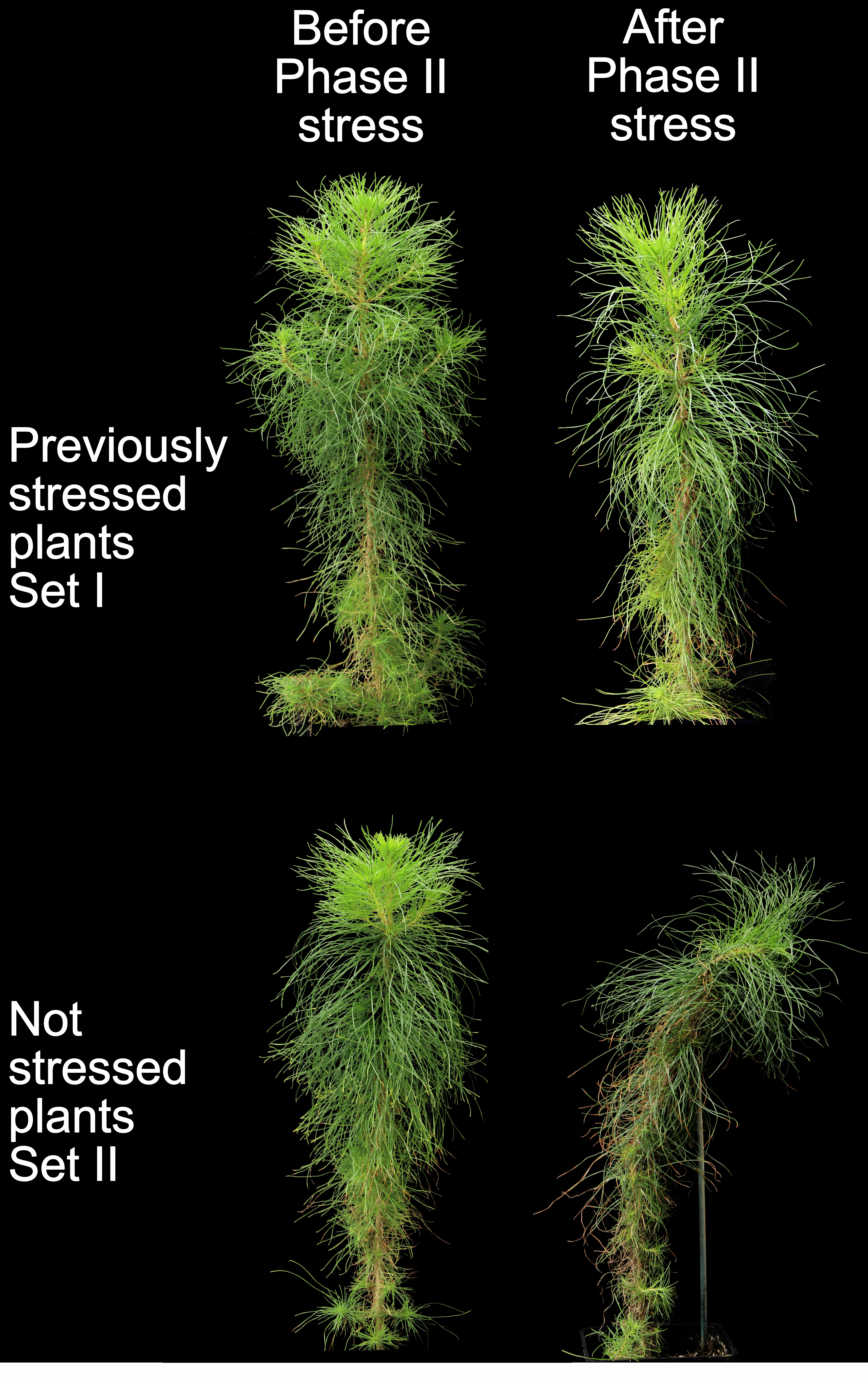

Figure S6: Representative pine seedlings from Set I (previously stressed) and Set II (not previously stressed) before and after Phase II stress corresponding to SC, ST5, NSC and NST5 sampling points. Apical bud of not primed plants showed a decay after the stress exposure (NST5) along with damaged needle tips and dry needles, while previously stressed plants (ST5) manifested no signs of severe impairment, showing damage only in few needle tips.
